# Promoting the Calcium-Uptake Bioactivity of Casein Phosphopeptides *in vitro* and *in vivo*

**DOI:** 10.3389/fnut.2021.743791

**Published:** 2021-08-30

**Authors:** Guo Liu, Baoyan Guo, Shengwei Sun, Minna Luo, Fei Liu, Jianyin Miao, Jian Tang, Yahui Huang, Yong Cao, Mingyue Song

**Affiliations:** ^1^Guangdong Provincial Key Laboratory of Nutraceuticals and Functional Foods, College of Food Science, South China Agricultural University, Guangzhou, China; ^2^College of Horticulture, South China Agricultural University, Guangzhou, China; ^3^Guangzhou Greencream Biotech Co., Ltd., Guangzhou, China; ^4^Infinitus (China) Company Ltd., Guangzhou, China

**Keywords:** casein phosphopeptides, calcium binding capacity, calcium uptake bioactivities, Caco-2, animal model

## Abstract

Casein phosphopeptides have been studied widely for their ability to chelate calcium. However, systematic studies on the effects of casein phosphopeptides (CPP) on calcium absorption *in vitro* and *in vivo* are scarce. The purities of two commercially available products, CPP1 and CPP2, are 18.37 and 25.12%, respectively. Here, the *in vitro* calcium binding capacity of CPP2 was 142.56 ± 7.39 mg/g, which was higher than that of CPP1 (107.15 ± 6.27 mg/g). The calcium transport results in a Caco-2 monolayer model indicated that, relative to controls, CPP1 and CPP2 increased calcium transport by 21.78 and 53.68%, respectively. Subsequent animal experiments showed that the CPP2-Ca-H group (1% Ca, 0.4% CPP2) had significant increases in the femur index, serum Ca^2+^ and serum osteocalcin levels, and femoral Ca content. The CPP2-Ca-H animal also had decreased serum alkaline phosphatase levels, parathyroid hormone content, and urinary pyridinoline content. Overall, our results demonstrated that CPP2 had stronger effects on promoting calcium uptake than CPP1.

## Introduction

The element calcium is an essential mineral nutrient for maintaining the good health of humans (1). In the human body, calcium accounts for ~1–2% of body weight. Of that, about 99% of calcium in humans is found in teeth and bones in the form of phosphate, where it contributes to overall structure and hardness (1). Calcium plays a crucial role in the body and participates in many biological processes such as cellular signal transduction, enzyme activity regulation, nerve conduction, and muscle contraction (2). It had been reported that calcium deficiency increases bone resorption and decreases bone mass (3). It has also been shown to be associated with development, including disorders such as rickets, osteoporosis (4), osteopenia, arterial hypertension, and kidney stones (5).

In recent years, ionized calcium has become the most common form of calcium available to humans (6). However, it is prone to forming calcium phosphate precipitation when it becomes ionized calcium in the basic environment provided by the intestine (7, 8). As a result, the low bioavailability of dietary ionized calcium severely restricts its usage. Consequently, the development of a better form of calcium that can overcome low absorption and bioavailability has become an important research topic.

Both amino acid calcium complex and small peptide calcium chelate can be directly absorbed in the human body (8). Currently, some calcium binding peptides have been developed from animal, aquatic, and plant food sources, (6) including duck egg peptides (9, 10), fish bones (1, 11), whey protein peptides (12, 13), and cucumber seed peptides (14).

Casein phosphopeptides are phosphorylated peptides that are derived from the enzymatic hydrolysis of casein. They can be produced from the digestion and hydrolysates of α_s1−_, α_s2−_, and β-casein *in vitro* and *in vivo* (15, 16). Casein phosphopeptides possess the capacity to chelate Ca^2+^ and avoid the precipitation of calcium phosphate salts. Due to this, they have been considered desired mineral carriers that have a potential role in strengthening elemental mineral absorption, which can, in turn, promote good health (17).

Although there has been much work in investigating casein phosphopeptides in recent years, few studies have systematically examined *in vitro* and *in vivo* calcium absorption. Accordingly, we sought to both characterize casein phosphopeptides and evaluate their *in vitro* and *in vivo* calcium absorption efficiencies. As such, their calcium binding capacities were examined in a sodium phosphate buffer, the calcium transport in Caco-2 cell monolayer model, and the bone metabolism in an animal model. This study not only provides basic theoretical research for the evaluation of casein phosphopeptides but also provides useful information for the optimization of production processes.

## Materials and Methods

### Materials

Infinitus (China) Company Ltd. (Guangzhou, China) donated two kinds of commercially available CPP mixtures (CPP1 and CPP2) for use in these experiments. Nitric acid and chromatographic-grade acetonitrile were bought from Merck (Merck KGaA, Darmstadt, Genmany). Cell culture media, penicillin, streptomycin, non-essential amino acids, Hank's balanced salt solution (HBSS) without Ca^2+^ and Mg^2+^, and fetal bovine serum were procured from Sigma (St. Louis, MO, USA). All other reagents were of an analytical grade.

### Composition of CPP

The contents of phosphorus, nitrogen, and purity in the commercial casein phosphopeptides (CPP) samples were determined according to the Chinese standard methods GB 5413.22-2010, GB 5009.5-2010, and GB 31617-2014, respectively. The nitrogen/phosphorus (N/P) molar ratio for each CPP mixture was established by dividing the nitrogen molar mass fraction by that for phosphorus. The CPP samples (1–2 g) were then weighted in an oven at 105°C to a consistent weight to calculate overall moisture content. Separate samples were dissolved in deionized water (10 mg/ml) to determine pH with a standard pH meter (Delta 320, Mettler Toledo, Switzerland).

### High-Performance Liquid Chromatography Analysis

The CPP1 and CPP2 high-performance liquid chromatography (HPLC) analyses of samples were performed using a Shimadzu LC-15C system with a reversed phase HPLC column [Diamond C18 (2), 5 μm, 4.6 mm, Dikma, China]. The elution condition was chosen based on a previously published method with minor changes (18). Briefly, solution A was 0.1% trifluoroacetic acid in deionized water, and solution B was 0.1% trifluoroacetic acid in acetonitrile. The flow rate was 1 ml/min. A linear gradient of solution B was applied as follows: 5–18% for 19.5 min, 18–30% B for 30 min, 90% B for 10 min, and 5% B for 10 min. Samples were filtered (0.22 μm) before injection. The injection volume was generally 20 μl. The elution was monitored at 215 nm.

### Calcium Binding Capacity in PBS Buffer

The calcium binding capacity was defined as the calcium amount (mg) bound to CPP (g) after chelation. This capacity was measured according to the previously published procedure with minor modifications (13, 18). Briefly, samples of CPP were made by dissolving it in 0.02 M of sodium phosphate buffer (pH 7.8) and were subsequently mixed with 5 mM of CaCl_2_. The solution was agitated at 37°C for 30 min at a constant pH (7.8) and then centrifuged at 4,000 rpm at 25°C for 20 min. The calcium content in the supernatant was quantitatively determined using inductively coupled plasma optical emission (ICP-OES) (ICPE 9820, Shimadzu, Kyoto, Japan).

### Caco-2 Cell Monolayer Establish and Calcium Transport Studies

Cell culture and calcium transportation were performed following our previously reported method (18, 19). Briefly, Caco-2 cell lines (50–60 passages) were used as a representative system in this study. Cells were cultured in cell culture flasks (Corning Inc., Tewksbury, MA, USA). The culture solution was complete Dulbecco's modified essential medium mixed with 10% fetal bovine serum, 1% non-essential amino acids, and 1% antibiotic. Cells were transferred into a CO_2_ incubator and then incubated at 37°C in 5% CO_2_. The culture medium was changed every 2 days until cultures were 90% confluent. Cells were detached from the incubator using trypsin and seeded on a six-well transwell plate with a polycarbonate membrane (24-mm diameter inserts, 0.4-μm pore size) at a density of 3 × 10^5^ cells/ml. The volume of the culture medium in the basolateral side and the apical sides was 3.5 and 2.5 ml, respectively.

The volume of the culture medium was 3.5 ml in the basolateral side and 2.5 ml in the apical sides. The culture medium was changed every 2 days for all 21 days of incubation. To assess the monolayer tight junction permeability, the values of transepithelial electric resistance (TEER) in the Caco-2 cell monolayer model were measured during whole experiment *via* a Millicell ERS-2 epithelial voltammeter (World Precision Instruments, Sarasota, FL, USA). Only when the TEER value is greater than 250 Ω•cm^−2^ would this model be considered successful and allowed to proceed to calcium transport experiments.

The monolayer culture medium was first removed, and then, cells were washed twice using HBSS without calcium and magnesium that was preheated at 37°C. Caco-2 cell monolayers in the transwell insert were immediately transferred to a new six-well plate. Two milliliters of the HBSS buffer was added into the basolateral side and the apical side. The entire transport experiments were carried out at 37°C in 5% CO_2_ except for the addition of samples. All samples used in the calcium transport experiments were dissolved in HBSS. The CPP1 and CPP2 (100 μg/well) and calcium solution (300 μg/well) were pre-blended immediately prior to the start of the experiment and then added to the apical sides. The calcium level was 300 μg/well in the control group. All cells were incubated for 0, 20, 40, 60, 90, 120, 180, and 240 min, and 1 ml of basolateral solution was collected at each of these time points to assess Ca^2+^ levels. After the removal of each 1 ml-sample at the designated time point, the same volume of a fresh HBSS buffer was immediately added to the basolateral side to maintain the integrity of the calcium transport system.

The harvested, time-point-specific basolateral solution samples were digested with aqua regia and diluted using deionized water. The concentration of Ca^2+^ was subsequently measured using an ICP-mass spectrometry (ICP-MS) (NexION 300X ICP, Waltham, MA, USA). These data were then used to calculate the total transported Ca^2+^ using the previously reported equation (18):

$$\begin{array}{l} B_{n}=2\times A_{n}+1\times \overset{n-1}{\underset{k-1}{\sum }}A_{k} \end{array}$$

where B_n_ denotes the total transported Ca^2+^ on the basolateral side of each well at each sampled time (0, 20, 40, 60, 90, 120, 180, and 240 min), 2 is a constant representing the 2 ml of HBSS buffer on the basolateral side, A_n_ indicates the Ca^2+^ concentration in collected basolateral HBSS buffer of each well at each sampled time, 1 indicates the 1 ml of basolateral HBSS buffer collected from each well at each sampled time, and n denotes the collection times of the basolateral solution samples.

### Animals and Diets

All the experiments were performed at the South China Agricultural University animal science center and then followed the South China Agricultural University institutional guidelines [SYXY (Yue) 2014-0136]. All test animal programs were strictly performed in conformity with the Chinese Association for Laboratory Animal Sciences and ratified by the Ethical Committee for Animal Experimentation of the South China Agricultural University.

In the animal test, 48 male SD rats (3 weeks old) were purchased from the Guangdong Medical Experimental Animal Center. Rats were individually housed in rearing cages in a Specific Pathogen Free grade room, at 23 ± 2°C and 40–75% humidity (12-h light/dark) at the Animal Center in the South China Agricultural University. Deionized water was used as the drinking water for all the animals. The diet was prepared according to the AIN-93M (20) with either normal calcium (5-g Ca/kg diet, normal diet) or supplemented calcium (10-g Ca/kg diet, supplemented calcium diet) with an additional low dose or high dose of one of two commercially available CPP (low dose 0.2%, high dose 0.4%).

### Feeding Procedures

After a 1-week habituation period, all rats were randomly segregated into six groups according to body weight. These six groups were as follows: control, CaCO_3_, two different CPP1 doses with calcium (two groups), and two different CPP2 doses with calcium (two groups). There were eight rats per group. The CaCO_3_ group was fed with the supplemented calcium diet (1% Ca), the CPP1-Ca-L group was fed with the supplemented calcium and low-dose CPP1 diet (1% Ca, 0.2% CPP1), the CPP1-Ca-H group was fed with the supplemented calcium and high-dose CPP1 diet (1% Ca, 0.4% CPP1), the CPP2-Ca-L group was fed with the supplemented calcium and low-dose CPP2 diet (1% Ca, 0.2% CPP2), the CPP2-Ca-H group was fed with the supplemented calcium and high-dose CPP2 diet (1% Ca, 0.4% CPP2), and the control group was fed with the normal diet (0.5% Ca). The six experiment groups were fed with their respective diets for seven continuous weeks.

### Sampling and Analytic Methods

The daily diet feed intakes and weekly body weights of all the groups were recorded routinely. Urine was collected from all rats during the last 3 days using a metabolic cage before feeding and euthanizing. To remove any contaminating sediments, the urine was centrifuged at 4,000 g for 10 min at 4°C to obtain the supernatant. Urinary pyridinoline content was measured using a commercially available ELISA Kit according to the instructions of the manufacturer (Nanjing Jiancheng Bioengineering Institute, China).

After the 7-week feeding period, all rats were anesthetized with diethyl ether. Suborbital blood was collected by removing the eyeball. After a 30-min stand still, the samples were centrifuged at 1,000 g and 4°C for 30 min. The Ca^2+^, osteocalcin (OCN), parathyroid hormone (PTH), and alkaline phosphatase (ALP) levels in the serum were analyzed using a commercially available ELISA Kit and according to the Jincheng instructions of the manufacturer (Nanjing Jiancheng Bioengineering Institute, China). Rats were then sacrificed by rapid cervical dislocation, and the organs (heart, liver, spleen, lung, and kidney) of each rat were collected to calculate the visceral index. The length of the femur was measured by a caliper. Any tissue adhering to the femur was removed. The weight of the femur was weighted using a balance (AL104, Mettler Toledo, Switzerland). The bone mineral density (BMD) of the femur in each group was assayed *in vitro* using dual-energy X-ray absorptiometry (Discovery A S/N 82239, HOLOGIC, Marlborough, MA, USA). Femurs were dried at 105°C for 24 h and then ground into a fine powder using a mortar. The resulting powder was digested using an acid mixture (nitric acid and perchloric acid at 4:1 ratio) and then dissolved in 5% nitric acid. Calcium levels were subsequently analyzed using ICP-OES (10-ES, Varian, Palo Alto, CA, USA).

### Statistical Analysis

All results in this study were presented as mean ± SD. All statistics analyses were performed using IBM SPSS Statistics (version 17). The ANOVA model was used for the comparison of differences among three or more groups. A *P* < *0.05* was considered statistically significant.

## Result and Discussion

### Composition of Two CPP Samples

Table 1 shows the composition results of the commercially available CPP1 and CPP2. The purity of the CPP1 and CPP2 was 18.37 and 25.12%, respectively. The results revealed that the purity of CPP2 was much higher than that of CPP1 after optimizing the production method. In this study, the N/P value for CPP1 (65.89 ± 3.5%) was significantly higher than that of CPP2 (35.14 ± 2.1%). It was reported that CPP purity was expressed by the nitrogen/phosphate (N/P) molar ratio, the lower N/P molar ratio, and the higher purity of CPP (21). The results of the N/P molar ratio were in line with the purity of CPP1 and CPP2. The moisture contents were 2.98 and 3.93%, respectively.

**Table 1:** Composition of two commercially available casein phosphopeptides (CPP).

	**CPP1**	**CPP2**
Purity (%)	18.37 ± 1.6^a^	25.12 ± 2.2^b^
Nitrogen content (g/100 g)	13.55 ± 0.2^a^	15.36 ± 0.3^b^
Phosphorus content (g/100 g)	0.46 ± 0.1^a^	0.98 ± 0.1^b^
N/P	65.89 ± 3.5^b^	35.14 ± 2.1^a^
Moisture content (%)	2.98 ± 0.1^a^	3.93 ± 0.1^b^
pH	5.94 ± 0.1^b^	5.56 ± 0.1^a^

*All data are presented as the means ± SD of three independent experiments. The different letters in the bar chart were considered statistically significant differences between groups according to Student's t-test (P < 0.05)*.

### CPP2 Had More Levels of Weak Polar Peptides Compared With CPP1

**Figure 1:**
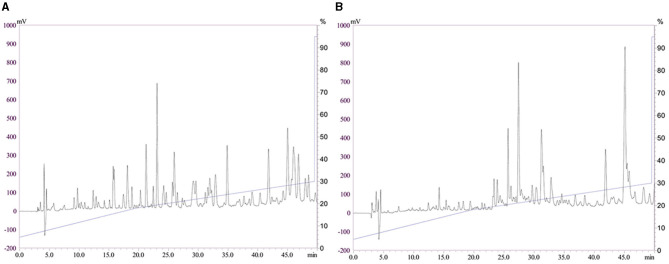
Reversed phase high performance liquid chromatography profiles of casein phosphopeptides 1 and casein phosphopeptides 2 [CPP1 **(A)** and CPP2 **(B)**].

The HPLC profiles of CPP1 and CPP2 are shown in Figures 1A,B. The samples were separated on an RP-C18 HPLC column, and a number of peaks for CPP1 and CPP2 were observed. When compared with CPP1, CPP2 had relatively smaller peaks between the 5th and the 20th min. The concentration of acetonitrile in the organic solvent mobile phase gradually increased as time went on, with the weak polar peptides being eluted later. Based on our results, CPP2 had a lower content of strong polar components when compared with CPP1, which suggest that optimizing the production method removed the strong polar peptides and reserved the weak polar peptides.

### Composition of CPP

The HPLC profiles of CPP1 and CPP2 are shown in Figures 1A,B.

**Figure 2:**
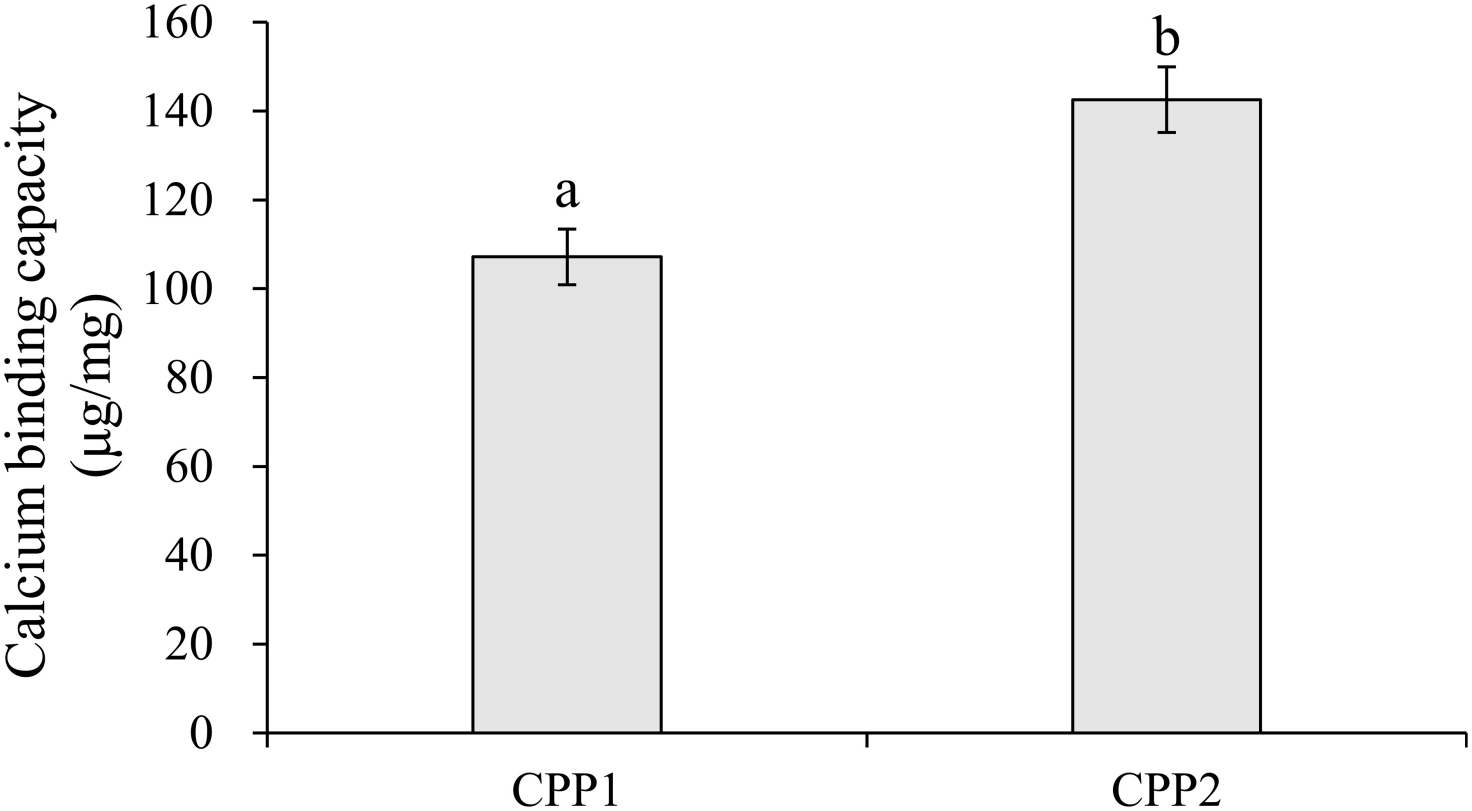
The calcium binding capacity of CPP1 and CPP2. All data are presented as the means ± SD of three independent experiments. Different letters indicate statistical difference among the groups at the given time point according to Student's t test (*P* < 0.05).

Compared to CPP1, CPP2 significantly increased calcium binding capacity *in vitro*. The calcium chelating capacities of CPP1 and CPP2 are shown in Figure 2. The calcium binding capacities of CPP1 and CPP2 were 107.15 ± 6.27 and 142.56 ± 7.39 mg/g, respectively. The calcium binding value of CPP2 was ~1.33-fold higher when compared with that of CPP1, indicating that CPP2 could chelate more calcium than CPP1. The reason for the above difference in calcium binding capacities was due to the lower purity and higher N/P molar ratio of CPP1 when compared with CPP2. The relationship we observed between the N/P values (Table 1) and calcium binding capacity was in agreement with published reports (22). Taken together, these data showed that calcium binding ability increased with decreasing N/P molar ratio.

### CPP2 Significantly Improved Calcium Transport Compared to CPP1 in the Caco-2 Cell Monolayer

**Figure 3:**
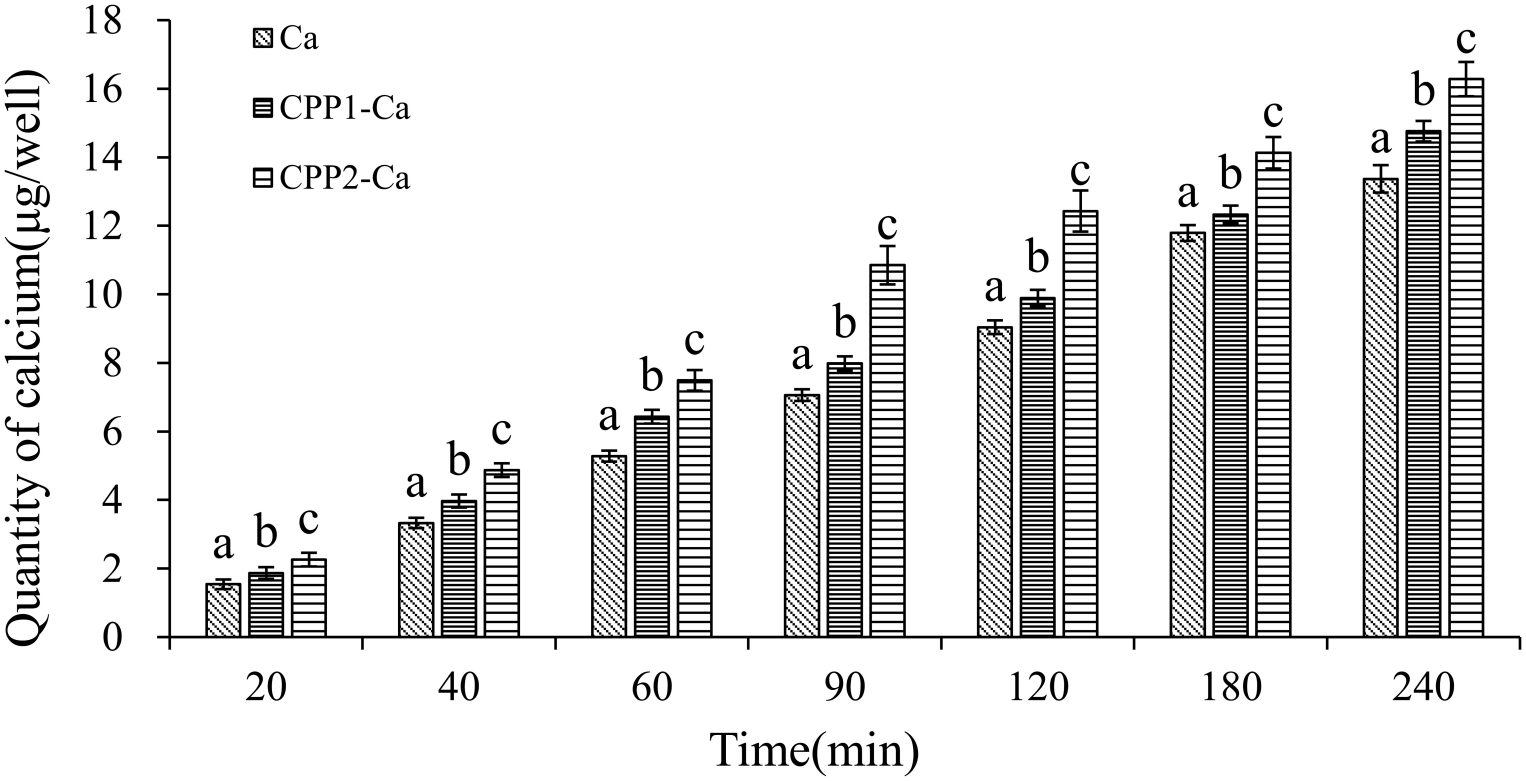
Quantity of calcium transport across Caco-2 cell monolayers by CPP1 and CPP2. Different letters indicate statistical difference among the groups at the given time-point (*P* < 0.05).

During the 21 days of cell culture, Caco-2 cells underwent a series of spontaneous differentiations to the formation of a monolayer of cells (23). This layer possessed the morphological and functional features of mature enterocytes, such as the microvillar structures, several types of brush-border enzymes, exopeptidases, alkaline phosphatase, a carrier-mediated transport system for di- or tripeptides, and so on (23). The Caco-2 cell monolayer model has been successfully applied to simulated absorption studies involving mineral elements (24). In this study, CPP1 and CPP2 calcium transport activities were assessed. Both CPP1 and CPP2 could significantly improve calcium transport activity in the Caco-2 monolayer model when compared with CPP control. As shown in Figure 3, this improvement was evident across all values from the 20 to 240 min time points (*P* < *0.05*). The rate of CPP1 calcium transport values was high in the first 60 min, after which, calcium transport proceeded slowly. The rate of calcium transportation of CPP1 was ~1.21-, 1.19-, 1.22-, 1.13-, 1.09-, 1.05-, and 1.10-fold higher than the control at 20, 40, 60, 90, 120, 180, and 240 min. Similar with the trend seen with CPP1, the rate of CPP2 calcium transport was high during the first 90 min before it proceeded to slow. The rate of calcium transportation of CPP2 was ~1.47-, 1.46-, 1.42-, 1.54-, 1.38-, 1.20-, and 1.21-fold higher than the control from the 20 to 240 min time-points, respectively. Moreover, CPP2 increased calcium transport by 21, 23, 16, 35, 27, 15, and 10% relative to CPP1 values at corresponding time-points (from 20 to 240 min, respectively).

There are two pathways that might be responsible for intestinal calcium absorption, namely, the paracellular movement and transcellular pathways (25, 26). Various mechanism studies have reported the effect of bioactive peptides on intestinal calcium absorption. For example, a study showed that the main way of calcium transport across the small intestine and Caco-2 cell monolayers was through a paracellular way (27, 28). Perego et al. also indicated that increased calcium uptake by the peptide may be due to the interaction of the peptide with either (1) the TRPV6 calcium channel or (2) voltage-operated L-type calcium channels to activate calcium entry into the cells (29, 30). Hou et al. used desalted duck egg white peptides using the Caco-2 cell monolayer model to show that the paracellular pathway was a minor contributor to calcium transport (10). Collectively, this study indicated that multiple mechanisms may exist in the calcium uptake mediated by peptides with different molecular structures (31).

Calcium and CPP effects on cellular viability and monolayer integrity were monitored between all samples and control using TEER values throughout the entirety of the experiment. As shown in Table 2, sample TEER values are not significantly different from controls.

**Table 2:** Transepithelial electric resistance (TEER) values of the cell monolayers in between the samples and control during the whole experimentation.

**Groups**	**Cultured for 21 days in DMEM and begin transporting (Ω·cm^**–2**^)**	**Cultured for 30 min in HBSS buffer (Ω·cm^**–2**^)**	**Complete transporting after 240 min in HBSS buffer (Ω·cm^**–2**^)**
Blank control	255 ± 5^a^	160 ± 7^a^	151 ± 7^a^
Calcium control	250 ± 4^a^	163 ± 4^a^	146 ± 8^a^
CPP1	248 ± 5^a^	158 ± 6^a^	154 ± 5^a^
CPP2	247 ± 7^a^	156 ± 8^a^	153 ± 7^a^

*Mean ± SD (n = 3). Different letters indicate statistical difference among the groups to TEER (P < 0.05). Blank control: no addition of calcium or samples CPP. Calcium control: only added calcium (300 μg/well) and no CPP. CPP1: addition of calcium and CPP1. CPP2: addition of calcium and CPP2*.

### No Significant Differences Were Found in Weight Gain and Visceral Index of All Groups

**Figure 4:**
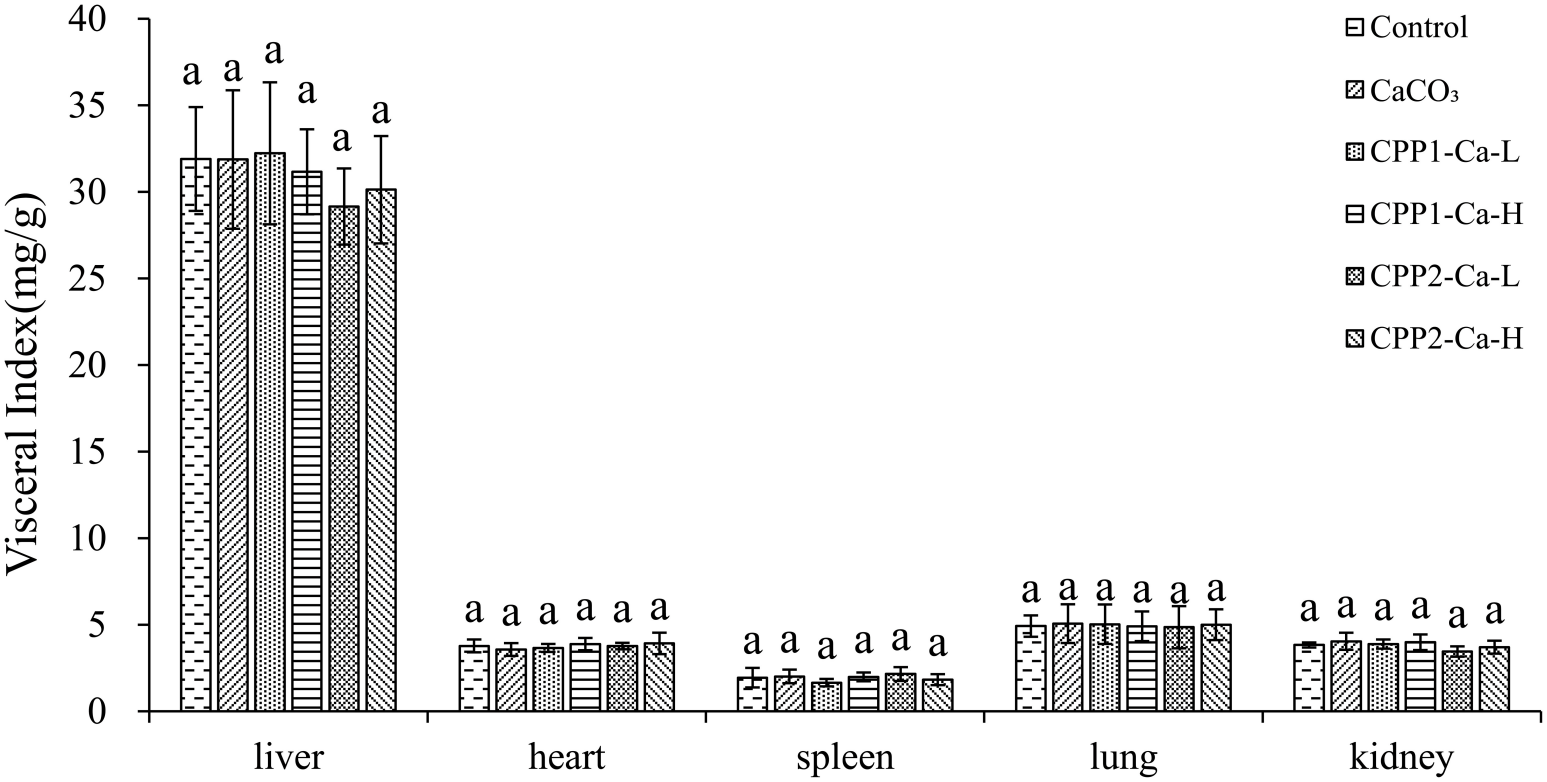
Visceral index of different viscus in different groups. Control group was fed a normal diet, and CaCO_3_ group was fed a supplemented calcium diet (1% calcium). CPP1-Ca-L, CPP1-Ca-H, CPP2-Ca-L, and CPP2-Ca-H were fed supplemented calcium with low-dose CPP1 (1% calcium, 0.2% CPP1), high-dose CPP1 (1% calcium, 0.4%), low-dose CPP2 (1% calcium, 0.2% CPP1), high-dose CPP2 (1% calcium, 0.4%), respectively. Different letters indicate statistical difference among the groups at the given time point (*P* < 0.05).

In this experiment, diarrhea was not observed during the entire experimental period. The visceral indices of different rat viscera (heart, liver, spleen, lung, and kidney) obtained from control, CaCO_3_, and CaCO_3_ with CPP groups are shown in Figure 4. Data regarding initial and final body weights and body weight gain were shown in Table 3. There were no significant differences in visceral indices and initial or final body weight gain in all the groups. These data revealed that CaCO_3_ and CaCO_3_ in CPP groups would not affect normal growth and health.

**Table 3:** Growth, femur, and biochemistry properties of rats after different treatments.

**Measurements**	**Control**	**CaCO_**3**_**	**CPP1-Ca-L**	**CPP1-Ca-H**	**CPP2-Ca-L**	**CPP2-Ca-H**
**Diet and growth**						
Initial weight (g)	170.2 ± 13.08^a^	166.58 ± 11.35^a^	162.95 ± 8.5^a^	171.5 ± 9.11^a^	168.6 ± 3.49^a^	166.2 ± 8.48^a^
Final weight (g)	787.2 ± 22.08^a^	739.33 ± 45.87^a^	791.45 ± 48.5^a^	785.23 ± 19^a^	752.58 ± 30.44^a^	773.6 ± 51.4^a^
Body weight gain (g)	617 ± 28.45^a^	572.75 ± 38.89^a^	628.5 ± 41.07^a^	613.73 ± 16.7^a^	583.98 ± 29.2^a^	607.4 ± 54.78^a^
Serum and unrinry biochemistry						
Serum Ca (mmol/L)	1.62 ± 0.02^a^	1.66 ± 0.03^a^	1.4 ± 0.69^a^	1.42 ± 0.68^a^	1.73 ± 0.05^b^	1.74 ± 0.04^b^
Alkaline phosphatase (U/ml)	242.35 ± 20.82^b^	173.56 ± 20.29^a^	186.36 ± 20.45^a^	184.26 ± 21.79^a^	188.64 ± 9.72^a^	185.75 ± 22.93^a^
Osteocalcin (ng/ml)	4.22 ± 0.22^a^	4.88 ± 0.38^a^^b^	4.85 ± 0.05^a^^b^	5.28 ± 1.38^a^^b^	4.89 ± 1.05^a^^b^	6.02 ± 0.15^b^
Parathyroid hormone (pg/ml)	318.59 ± 9.03^b^	297.78 ± 77.69^a^^b^	271.62 ± 23.47^a^^b^	265.21 ± 9.69^a^	280.78 ± 9.5^a^^b^	263.27 ± 11.02^a^
Urinary pyridinoline content (nmol/L)	62.64 ± 6.37^b^	55.77 ± 9.63^a^^b^	53.09 ± 4.37^a^^b^	56.2 ± 5.62^a^^b^	52.67 ± 2.52^a^^b^	46.22 ± 8.62^a^
Femur physicochemical indices						
Length (cm)	3.26 ± 0.16^a^	3.36 ± 0.11^a^	3.44 ± 0.07^a^	3.42 ± 0.06^a^	3.42 ± 0.06^a^	3.46 ± 0.08^a^
Weight (g)	1.35 ± 0.16^a^	1.31 ± 0.03^a^	1.4 ± 0.2^a^	1.45 ± 0.16^a^	1.43 ± 0.08^a^	1.38 ± 0.05^a^
Femur index	6.52 ± 0.41^a^	6.98 ± 0.21^a^^b^	7.12 ± 0.69^a^^b^	7.28 ± 0.13^a^^b^	7.43 ± 0.45^b^	7.43 ± 0.5^b^
Bone mineral density (g/cm^2^)	0.24 ± 0.01^a^	0.24 ± 0.01^a^	0.25 ± 0.01^a^	0.25 ± 0.01^a^	0.25 ± 0.01^a^	0.26 ± 0.01^a^
Ca content (mg/g)	105.54 ± 3.69^a^	115.49 ± 1.44^a^^b^	120.17 ± 4.69^a^^b^	119.72 ± 5.93^a^^b^	125.94 ± 4.56^b^	129.35 ± 9.32^b^

*Means ± SD (n = 8). Different letters indicate statistical difference among the groups (p < 0.05). The control group and CaCO_3_ group data is from the previous study (19)*.

### High Dose of the CPP2 Group Had a Significant Increase in the Serum Levels of Ca and OCN and a Decrease in the Serum ALP, PTH, and Urinary PYD Content

Alkaline phosphatase (ALP), as a biological marker for bone formation, plays a major role during the process of bone calcification, and its expression has been shown to be elevated in case of abnormal calcification (32). Osteocalcin (OCN) is another marker of bone formation and can be used as a biochemical index to evaluate the production of bones (33). As shown in Table 3, the serum ALP levels are significantly lower in groups treated with just CaCO_3_ and CaCO_3_ supplemented with CPP groups when compared with the controls (*P* < 0.05), while there was no significant difference between CaCO_3_ and CaCO_3_ with CPP groups. These results were similar to the recent findings demonstrating no significant difference between CaCO_3_ and CaCO_3_ supplemented with peptides obtained from the tilapia scale peptide (32) or cod (1). Serum OCN levels in CaCO_3_ and CaCO_3_ with CPP groups were increased when compared with the controls. Although there were no significant differences between the CaCO_3_ and CaCO_3_ with CPP groups, these results were consistent with the previous study (11). The CPP2-Ca-H group was significantly higher than the control group (*P* < 0.05). There were no significant differences in serum Ca^2+^ levels among control, CPP1-Ca-L, and CPP1-Ca-H groups. These findings were in agreement with the previous studies (11, 34, 35). However, the serum Ca^2+^ levels in CPP2-Ca-L and CPP2-Ca-H groups were significantly higher than those of controls. Taken together, these results indicated that both CPP1 and CPP2 could enhance bone formation, particularly under the conditions presented in the CPP2-Ca-H group.

Parathyroid hormone (PTH) plays an important role in sustaining calcium concentration in the blood. A high PTH concentration increases calcium release from the bone and increases bone resorption (36). In line with this, serum PTH content was measured and found to be lower in both the CaCO_3_ and CaCO_3_ with CPP groups when compared with controls. This difference was particularly apparent between control and CPP2-Ca-H groups. Deoxypyridinoline was a bone resorption marker and was a crossing of type I collagen present in bone (37). It was also called D-pyrilinks and determined through pyridinoline (PYD) level (38). Urinary PYD levels in all groups were found to be higher than that of the control group. In particular, there was a significant difference between CPP2-Ca-H group and the control group. Collectively, these results revealed that both CPP1 and CPP2 could decrease bone resorption particularly under the conditions presented in the CPP2-Ca-H group.

These results indicated that CPP1 and CPP2 provided in the diet could increase bone formation and prevent bone resorption. Of these, the condition presented by the CPP2-Ca-H treatment group was the most effective.

### High Dose of the CPP2 Group Significantly Increased the Femur Index and Femur Ca Content

Changes to femoral properties were an ideal indicator to monitor the effects of calcium supplementation in this experiment. This is because they are sensitive to Ca^2+^ assimilation and metabolism (39). In this experiment, no statistically significant differences in femoral weight and femoral length were found. These findings were similar to other studies, which found no significant increase in femoral length in subjects treated with the fish-bone peptide when compared with controls (35). Other studies have shown that the femoral and dry weight in low and medium gavage groups were not significantly different when compared with controls apart from the high-dose gavage group (11). To this end, rats fed with CaCO_3_ with CPP2 groups showed significantly higher Ca femoral content than controls (*P* < 0.05). This is in agreement with the previous findings of other studies (32). The BMD of the CPP-Ca-H group was higher than those of control and CaCO_3_ groups. Although there were no significant differences in femoral BMD among control, CaCO_3_, and CaCO_3_ with CPP groups, they exhibited a trend toward increasing BMD in all the groups that were fed with CPP1 and CPP2. One possible reason for this observation could be the low purity in CPP1 and CPP2 groups. A second possible reason could be that the feeding time was not long enough.

Taken together, the femoral property results illustrated that CPP1 and CPP2 can promote calcium incorporation into bone in rats. Specifically, the high dose of the CPP2 group was much more effective in promoting calcium absorption and bone calcification. In the results of the calcium binding ability experiment *in vitro*, the Caco-2 cell model and animal model all showed that CPP2 had better calcium binding ability and promoted greater calcium uptake and calcium absorption abilities than CPP1. Although the activity of the product CPP2 was significantly higher than that of CPP1, the functional properties were important factors affecting its application. In the future, the functional characteristics of the two CPP will be systematically studied.

## Data Availability Statement

The original contributions presented in the study are included in the article/supplementary material, further inquiries can be directed to the corresponding authors.

## Ethics Statement

The animal study was reviewed and approved by Committee of South China Agriculture University.

## Author Contributions

GL, YC, and MS designed the experiment. GL, BG, SS, ML, and FL conducted the animal experiments. GL, BG, SS, and FL conducted the cell experiments. GL, JM, YH, YC, and MS wrote and revised the manuscript. GL, BG, SS, ML, and JT did the experimental analysis, collection, and analysis of the data. All authors contributed to the article and approved the submitted version.

## Conflict of Interest

FL is employed by Guangzhou Greencream Biotech Co., Ltd. JT is employed by Infinitus (China) Company Ltd. The remaining authors declare that the research was conducted in the absence of any commercial or financial relationships that could be construed as a potential conflict of interest.

## Publisher's Note

All claims expressed in this article are solely those of the authors and do not necessarily represent those of their affiliated organizations, or those of the publisher, the editors and the reviewers. Any product that may be evaluated in this article, or claim that may be made by its manufacturer, is not guaranteed or endorsed by the publisher.
